# Functionalized Keratin as Nanotechnology-Based Drug Delivery System for the Pharmacological Treatment of Osteosarcoma

**DOI:** 10.3390/ijms19113670

**Published:** 2018-11-20

**Authors:** Elisa Martella, Claudia Ferroni, Andrea Guerrini, Marco Ballestri, Marta Columbaro, Spartaco Santi, Giovanna Sotgiu, Massimo Serra, Davide Maria Donati, Enrico Lucarelli, Greta Varchi, Serena Duchi

**Affiliations:** 1 Institute of Organic Synthesis and Photoreactivity (ISOF), National Research Council (CNR), Via Gobetti, 101, 40129 Bologna, Italy; elisa.martella@isof.cnr.it (E.M.); claudia.ferroni@isof.cnr.it (C.F.); andrea.guerrini@isof.cnr.it (A.G.); marco.ballestri@isof.cnr.it (M.B.); giovanna.sotgiu@isof.cnr.it (G.S.); greta.varchi@isof.cnr.it (G.V.); 2 Laboratory of Musculoskeletal Cell Biology, IRCCS Istituto Ortopedico Rizzoli, Via di Barbiano 1/10, 40136 Bologna, Italy; marta.columbaro@ior.it; 3 Institute of Molecular Genetics, National Research Council of Italy, 40136 Bologna, Italy; spartaco.santi@cnr.it; 4 IRCCS Istituto Ortopedico Rizzoli, Via di Barbiano 1/10, 40136 Bologna, Italy; 5 Laboratory of Experimental Oncology, IRCCS Istituto Ortopedico Rizzoli, Via di Barbiano 1/10, 40136 Bologna, Italy; massimo.serra@ior.it; 6 3rd Orthopaedic and Traumatologic Clinic prevalently Oncologic, IRCCS Istituto Ortopedico Rizzoli, Via di Barbiano 1/10, 40136 Bologna, Italy; davidemaria.donati@ior.it; 7 Department of Biomedical and Neuromotor Sciences (DIBINEM), Alma Mater Studiorum University of Bologna, Via Ugo Foscolo 9, 40123 Bologna, Italy; 8 Unit of Orthopaedic Pathology and Osteoarticular Tissue Regeneration, 3rd Orthopaedic and Traumatologic Clinic prevalently Oncologic, IRCCS Istituto Ortopedico Rizzoli, Via di Barbiano 1/10, 40136 Bologna, Italy; enrico.lucarelli@ior.it

**Keywords:** bone, musculoskeletal tumor, osteosarcoma, keratin, Photodynamic therapy, paclitaxel, Chlorin-e6

## Abstract

Osteosarcoma therapy might be moving toward nanotechnology-based drug delivery systems to reduce the cytotoxicity of antineoplastic drugs and improve their pharmacokinetics. In this paper, we present, for the first time, an extensive chemical and in vitro characterization of dual-loaded photo- and chemo-active keratin nanoparticles as a novel drug delivery system to treat osteosarcoma. The nanoparticles are prepared from high molecular weight and hydrosoluble keratin, suitably functionalized with the photosensitizer Chlorin-e6 (Ce6) and then loaded with the chemotherapeutic drug Paclitaxel (PTX). This multi-modal PTX-Ce6@Ker nanoformulation is prepared by both drug-induced aggregation and desolvation methods, and a comprehensive physicochemical characterization is performed. PTX-Ce6@Ker efficacy is tested on osteosarcoma tumor cell lines, including chemo-resistant cells, using 2D and 3D model systems. The single and combined contributions of PTX and Ce6 is evaluated, and results show that PTX retains its activity while being vehiculated through keratin. Moreover, PTX and Ce6 act in an additive manner, demonstrating that the combination of the cytostatic blockage of PTX and the oxidative damage of ROS upon light irradiation have a far superior effect compared to singularly administered PTX or Ce6. Our findings provide the proof of principle for the development of a novel, nanotechnology-based drug delivery system for the treatment of osteosarcoma.

## 1. Introduction

Osteosarcoma (OS) is the most common type of bone sarcoma diagnosed in childhood [1]. Neoadjuvant chemotherapy and surgery, followed by post-operative adjuvant chemotherapy, is the current standard treatment for OS [2]. Combined chemotherapy regimens are currently used in clinics [3], but they display diverse degrees of efficacy, which are largely dependent on the tumors’ stage [4,5] with a 5-year survival rate being below 40% for patients with lung metastasis at presentation [6]. The major drawbacks that hinder pharmacological treatment in OS patients are chemoresistance [7], and damage to key organs [8,9] (https://www.cancer.org/cancer/osteosarcoma/treating.html).

Therefore, considering the vast heterogeneity of OS molecular profiles [6] and the standard chemotherapeutic treatment limits, the future of a pharmacological cure for OS may be moving away from anti-oncogenic paradigms toward more effective and selective nanotechnology-based drug delivery systems (DDS). These systems represent a promising strategy for improving antineoplastic agents’ efficacy, pharmacokinetic profiles, and selectivity to the target [10,11,12,13].

Among various antitumor drugs, Paclitaxel (PTX) is one of the most effective, and is extensively used for treating breast, ovarian, lung, and pancreatic cancer [14,15]. However, the optimal clinical utility of PTX has been limited due to some critical issues, such as its very poor water solubility (0.3 μg/mL), which requires the use of highly toxic solvents, as a 1/1 mixture of Cremophor EL^®^/absolute ethanol (Taxol^®^) [16], with the consequent insurgence of severe side effects. The efficacy of Taxol for treating OS was formerly evaluated in two different trials for patients with high-grade OS and its variants [17], and for recurrent or metastatic soft-tissue sarcomas [18] displaying any significant success. In 2006, the FDA approved a new PTX formulation, the nab-paclitaxel (Abraxane™), where PTX is bound to human albumin in the form of nanoparticles [19]. This formulation provided interesting results in high-grade OS, with greater antitumor efficacy compared with the Taxol formulation itself, and was given also the ability of Abraxane™ to overcome ABCB1-mediated PTX resistance [20]. A comparative in vivo study demonstrated that the at high-dose of Abraxane^TM^ (e.g., 40 mg/kg) induced a tumor inhibitory rate of 98.8%, as compared to Taxol^®^ (40.8%) and Adryamicin (46.1%) [21]. These encouraging results indicate that nanotechnology-based DDS for PTX could represent a promising therapeutic option for OS treatment.

Even if Abraxane™ formulation provides several benefits over PTX in terms of enhanced water solubility, improved biodistribution profile, and decreased drug degradation and clearance, there are several data reporting its poor colloidal stability once injected into bloodstream, resulting in a PTX plasma concentration curve below the therapeutic window after few hours and a subsequent nonspecific biodistribution, similar to free PTX. [22]. To overcome these limitations, several materials, both synthetic and natural, are currently being extensively investigated as valid alternatives to become drugs carriers in cancer treatment and hence to improve drugs efficacy [23]. In the present work, we focused our attention on keratin. This natural biopolymer is a cysteine-rich structural protein, highly biocompatible and with exclusive tri-peptidic sequences present on its backbone, such as the “Arg–Gly–Asp” (RGD) and “Leu–Asp–Val” (LDV) sequences, that specifically bind vitronectin integrin receptors which are overexpressed by several cancer cells, including osteosarcoma cells [24]. Moreover, when compared to albumin, keratin displays higher drug-loading ability, yields, reproducibility, and easier scalability, due to its unique structural and chemical features [23,25]. All of these properties make keratin a promising material for nanoparticles-mediated drug delivery.

Alternative co-treatment techniques that do not require the use ofhighly toxic chemotherapeutics have been explored for enhancing the treatment outcome through a synergistic or additive effect. In this view, the use of photodynamic therapy (PDT) as a co-adjuvant therapy for cancer treatment is increasingly being considered [26,27]. PDT has been successfully used to kill OS cells in vitro [28,29,30,31,32,33,34] and in vivo [35,36,37]. In a previous study, we successfully demonstrated the in vitro efficacy of PDT in OS cell lines by using porphyrin-loaded poly-methyl methacrylate nanoparticles [38]. More recently, we reported the generation and the in vitro biological activity of keratin nanoparticles covalently loaded with the photosensitizer (PS) Chlorin-e6 (Ce6) [39]. These nanoparticles favour higherCe6 intracellular internalization when compared to free Ce6 towards OS tumor cells. Interestingly, an increasing number of studies report the combination of PDT and chemotherapy as a promising bimodal approach for improving anticancer therapeutic efficiency, with minimized side effects [40,41,42,43,44].

In the current study, high molecular weight and hydrosoluble keratin was selected for preparing nanoformulations functionalized with both PTX and Ce6 as a novel approach for the pharmacological treatment of OS. This novel bimodal nanoformulation that combines chemo and photodynamic therapies, was generated with the aim to specifically deliver PTX to cancer cells, while decreasing its general toxicity and scarce intracellular accumulation, and also to provide a second therapeutic agent to wipe out OS cells that might have survived the PTX or become chemo-resistant.

## 2. Results and Discussion

### 2.1. Physicochemical Characterization of Keratin Nanoformulations

**Figure 1:**
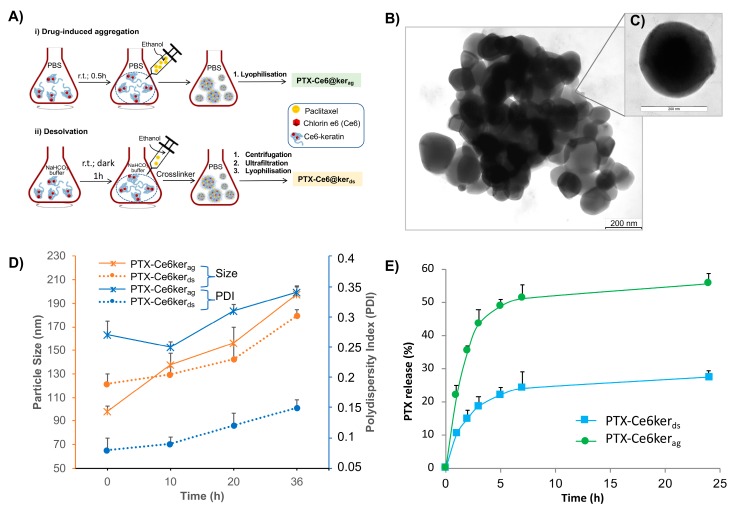
Synthesis and characterization on PTX-Ce6@ker nanoparticles. (**A**) Schematic representation of PTX-Ce6@ker synthesis through the (i) drug-induced aggregation (ag), and (ii) desolvation (ds) methods; (**B**,**C**) Trasmission Electron Microscopy (TEM) micrograph of PTX-Ce6@ker_ag_; (**D**) PTX-Ce6@ker_ag_ colloidal stability in Phosphate buffered solution (PBS) at 37 °C. Results are expressed as means of three independent experiments; (**E**) release profiles of PTX-Ce6@ker_ag_ and PTX-Ce6@ker_ds_ performed at 37 °C under stirring for 24 h. All results are expressed as the mean ± SD (from at least three independent experiments performed in triplicate).

To account for possible differences in PTX behavior in terms of release and efficacy over time, two different procedures were used for nanoparticle production: the drug-induced aggregation method (PTX-Ce6@ker_ag_), and the desolvation method (PTX-Ce6@ker_ds_) (Figure 1A).

In the aggregation method, the high affinity of PTX for keratin hydrophobic domains induces the spontaneous formation of stable nanoparticles [23], thus it does not require the use of toxic crosslinking agents and tedious purification procedures, such as ultrafiltration and dialysis. To maintain a fixed amount of Ker-Ce6, a double volume of PTX is required to provide PTX-Ce6@ker_ag_ formation. The nanoparticles produced with this method have an average hydrodynamic diameter of ∼150 nm, with a polydispersity index of 0.22 and a zeta-potential of −45.6 mV (Table S1). Moreover, since this procedure does not require any purification steps, the PTX loading ratio is 100%, since all the PTX used to induce the aggregation is loaded onto keratin nanoparticles.

In the desolvation method, the aggregation is induced by the denaturing solvent, that is, ethanol. Desolvated nanoparticles have an average hydrodynamic diameter of 120 nm, with a polydispersity index of 0.067 and a negative zeta-potential of −47.3 mV (Table S2). This procedure entails the use of much higher volumes of solvent and glutaraldehyde as a cross-linking agent, thus, it is expected that particles are smaller and more mono-disperse.

Ultrastructural studies performed by Trasmision Electron Microscopy (TEM) analysis show that these nanoparticles are spherical in shape with a smooth surface morphology and an average dry diameter of 115.6 ± 24.0 (Figure 1B).

Stability studies were performed in Phosphate Buffered Saline (PBS) at 37 °C (Figure 1C), indicating that a constant diameters’ increase (reported as size in the graph) of 50% and 37% occurred during the observation time for PTX-Ce6@Ker_ag_ and PTX-Ce6@Ker_ds_, respectively, due to a slight swelling of the particles. On the other hand, the nanoparticles’ polydispersity index (PDI in the graph) was not significantly affected during the observation time, which is in agreement with the satisfactory stability index of PTX-Ce6@Ker under these conditions. Overall, both nanoparticle formulations display similar trends of stability.

The single contributions of PTX and Ce6 were evaluated by preparing PTX@ker using both the desolvation (PTX@ker_ds_) and aggregation methods (PTX@ker_ag_), while the Ce6@ker particles were prepared exclusively by using the desolvation method [39], were and used as controls for all experiments.

The PTX release from nanoparticles is shown in Figure 1D. Both nanoparticles display a biphasic release trend, which includes an initial burst during the first 5 h, followed by a slower release in the following 24 h. The cumulative release during the 24 h was approximatively 55% and 27% for PTX-Ce6@Ker_ag_ and PTX-Ce6@Ker_ds_, respectively, suggesting that both particles can warrant prolonged effectiveness under physiological conditions. PTX-Ce6@Ker_ds_ was seen to display a lower release extent, which might be attributed to the lower matrix swelling for particles obtained by the desolvation procedure.

### 2.2. PTX Vehiculated through Keratin Nanoformulations Affect OS Cells’ Viability in 2D System

To account for OS tumor heterogeneity, the efficiency of the two nanoformulations was measured by a cytotoxicity test on three different OS tumor cell lines: MG63, SaOS-2, and U-2 OS.

To first assess the PTX activity, we measured the rate of cell death of free PTX and compared it with PTX loaded onto keratin.

**Figure 2:**
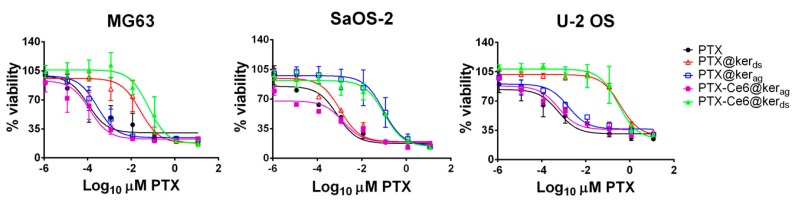
Sensitivity of osteosarcoma (OS) cell lines to free Paclitaxel (PTX), and PTX loaded onto keratin nanoparticles. The graphs show the WST-1 assay performed on OS cell lines exposed to PTX (black circles), PTX@ker_ag_ (blue squares), PTX@ker_ds_ (red triangles), PTX-Ce6@ker_ag_ (pink squares), or PTX-Ce6@ker_ds_ (green triangles). All results are expressed as the mean ± SD (from at least three independent experiments performed in triplicate).

The percentage of viability reported in the graphs of Figure 2 (see Table S3 for IC_50_ values), shows that free PTX and PTX@Ker/PTX-Ce6@ker, synthetized by both procedures and without light irradiation, affects all three cell lines, yet with different sensitivity. Notably, our data demonstrates that PTX retains its activity even once loaded onto keratin. This represents an essential feature for our nanotechnological delivery system, since the physiological response induced by the drug is not altered by keratin binding. In particular, MG63 cells were the most sensitive to the drug treatment, while SaOS-2 cells were the most resistant. Moreover, the three graphs highlight a marked difference in the IC_50_ profiles between the two nanoformulations, with the desolvated system showing a decrease in cell viability at higher concentrations compared to the aggregated formulation. In fact, as reported by Chen et al. [45], the crosslinking process (desolvation method) may affect the release of PTX or its efficacy by limiting drug diffusion in the interstitial spaces. On the other hand, the “not-crosslinked” formulation (drug-aggregation method), being less stable, might produce smaller aggregates that can more efficiently infiltrate through cells and ECM. Indeed, the PTX release profile was found to be consistent with the previous one (see Figure 1D). The different rate of PTX release from keratin could explain the different concentration of PTX efficacy in the two nanoformulations. Furthermore, the graphs show that the presence of Ce6 does not have a cytotoxic effect on all cell lines tested.

These preliminary data indicate that, while both nanoformulations are able to deliver PTX and affect OS cells’ viability, PTX-Ce6@ker_ag_ display a pharmacokinetic similar to free PTX. Moreover, the drug-induced aggregation method is more suitable for future applications in clinical settings, both in terms of easy scale-up and safety. Thus, all the following experiments were performed with the aggregated nanoformulation only.

### 2.3. The Localization of Keratin Nanoformulations in OS Tumor Cell Lines

**Figure 3:**
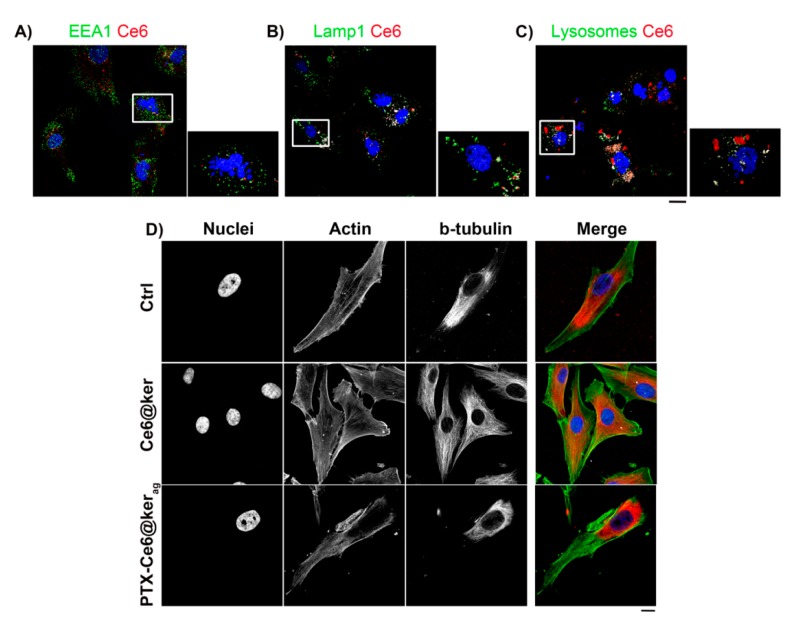
PTX-Ce6@ker_ag_ localization and the effect of PTX on MG63 cells. (**A**–**C**) Representative confocal images of MG63 treated for 24 h with PTX-Ce6@ker_ag_ and immunostained with EEA1, Lamp-1, and lysosomes, respectively (green channel in all pictures). The nuclei staining is shown in blue, the Ce6 signal in red, and colocalization in white; (**D**) Phalloidin (green channel) and β-tubulin (red channel) stainings were evaluated on MG63 at the end of the 24 h of treatment with Ce6@ker or PTX-Ce6@ker_ag_. Images are representative of at least three independent experiments. Scale bar: 20 µm.

In order to verify the localization of the nanoformulations once internalized by tumor cells, and the possible effect of the two drugs on cell morphology, a representative OS cell line, MG63, was exposed for 24 h in the dark to PTX-Ce6@Ker_ag_. The fluorescence of Ce6 was used as a readout for the intracellular detection of the nanoformulations, as shown in Figure 3 [39].

Immunostaining analyses, performed using markers of early and late endocytosis (EEA1 and Lamp-1, respectively) and lysosomes, revealed that right after the internalization (no light irradiation), only 12.2 ± 1.9% of the Ce6 signal was localized inside the vesicles of early endosomes (Figure 3A), while the colocalization percentage of the Ce6 signal with late endosomes was 23.7 ± 5.1% (Figure 3B), reaching the highest colocalization within lysosomes (41.5 ± 6.1%) (Figure 3C). Literature data account for a gradual decrease of Ce6 fluorescence intensity, at pH values typical of lysosomes (pH 4–5) [46]. Our data show that despite the covalent binding with keratin, no Ce6 fluorescence decrease takes place in organelles such as lysosomes, thus confirming the protective effect of the keratin protein over the PS.

The internalization of PTX-Ce6@Ker_ag_ into tumor cells at the end of the treatment (24 h) was confirmed by an altered cell morphology due to PTX inhibition of cell division via binding to microtubules’ cytoskeleton. Immunofluorescence analyses were performed on MG63 cells, which were untreated (Ctrl) or exposed to PTX-Ce6@ker and Ce6@ker, to discriminate between the effects due to chemotherapeutic drugs and Ce6. Interestingly, in the presence of PTX, the number of cells with multi-lobated nuclei significantly increased (80%) as compared to untreated cells (Ctrl), or cells treated with Ce6@Ker (Figure 3A,C, magnification panels), thus confirming the maintenance of PTX activity once loaded onto nanoparticles. Notably, the immunofluorescence analyses of MG63 treated in the dark with Ce6@ker shows that Ce6, by itself, does not have any effect on the cytoskeleton structure, while a complete alteration of the beta-tubulin organization can be observed when cells are treated with PTX-Ce6@ker_ag_ (Figure 3D). Taken together, these observations confirm that the activity of PTX is not altered once the drug is delivered by keratin.

### 2.4. Impact of PTX-Ce6@Ker_ag_ on OS Cells’ Viability in 2D System

To confirm the efficiency of the *ag* nanoformulation and to specifically assess the sequential contribution of PTX- and PDT-mediated treatments, OS cells’ viability was measured: (1) at the end of nanoparticle treatment in the dark (24 h) to evaluate PTX cytotoxicity, and (2) 24 h after light irradiation (using a LED source at 668 nm for 5 min) to evaluate Ce6 toxicity. MG63, SaOS-2, and U-2 OS cell lines were therefore treated for 24 h at three different dosages of PTX-Ce6@ker_ag_ nanoparticles, defined as PTX-Ce6@ker_ag_ (*low*), PTX-Ce6@ker_ag_ (*medium*), and PTX-Ce6@ker_ag_ (*high*) (Table 1).

**Figure 4:**
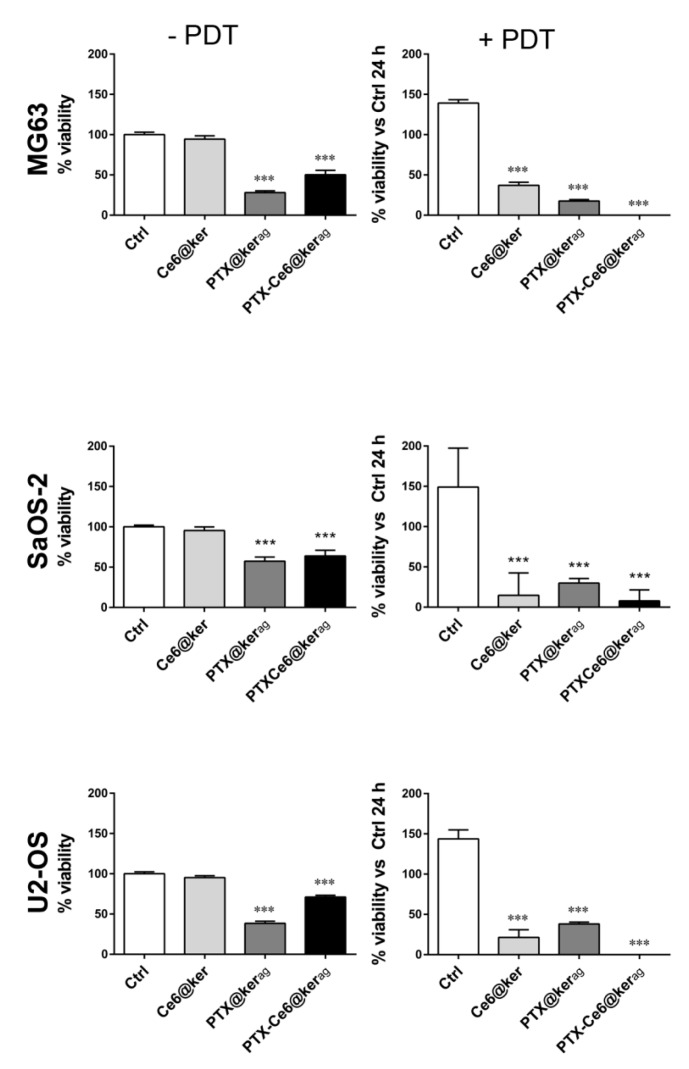
Impact of medium dosage of PTX-Ce6@Ker_ag_ on OS cells’ viability in a 2D system, and the additive effect of PTX and photodynamic therapy (PDT) on OS cell viability. OS cell lines were treated for 24 h with Ce6@ker, PTX@ker_ag_, or PTX-Ce6@ker_ag_ at medium concentration. The graphs show the Alamar blue assay performed immediately after keratin nanoparticle treatments (−PDT), and 24 h after irradiation of the same samples (+PDT). Data, normalized to untreated cells (Ctrl) at the first time-point, are expressed as the mean ± SD (*N* = 2 biological replicates; *N* = 3 technical replicates) and analyzed using the one-way ANOVA test, and Tukey’s multiple comparison test as a post-test. Results were considered to be statistically significant at *p* values < 0.05 (*** *p* values < 0.001).

As shown in the graphs in Figure 4, when cells were treated with the medium dosage, the impact of irradiation on nanoparticle-untreated tumor cells (Ctrl −/+PDT) is irrelevant on all cell lines used, supporting the hypothesis that light at that wavelength and fluence does not induce a cytotoxic effect by itself [39].

Any cytotoxicity can be detected in cells treated in the dark with Ce6@ker (Figure 4, Ce6@ker −PDT), while a strong reduction in cell viability can be observed in cells treated with Ce6@ker upon light irradiation (Figure 4, Ce6@ker +PDT). The effect of PTX on cell viability is statistically significant in all cell lines (PTX@ker_ag_, Figure 4 −/+PDT). The further decrease in cell viability observed on cells treated with PTX@ker_ag_ and stimulated with light, is most probably due to the long-term PTX effect after 24 h from PDT, since the light alone does not induce any cell damage in these samples where Ce6 is not present.

Notably, OS cells loaded with the multi-modal nanoparticle formulation upon light irradiation (PTX-Ce6@ker_ag_ +PDT) showed a dramatic decrease in cell viability, demonstrating that chemotherapy and photoactivation act in an additive manner, leading to massive cell death (100%) in all cell lines.

Similarly, we analyzed the cytotoxic effect on cells treated with nanoparticles at low and high dosages. The results indicate that with multi-modal nanoformulation at low concentrations, cell viability drops significantly, but does not reach the 100% level, as instead observed for the medium dosage (Figure S5), while at high concentrations, the photodynamic therapy has a predominant effect on cell viability, masking the additive effect of PTX activity (Figure S6).

### 2.5. Impact of PTX-Ce6@Ker_ag_ on Chemoresistant OS Cells’ Viability in 2D System

**Figure 5:**
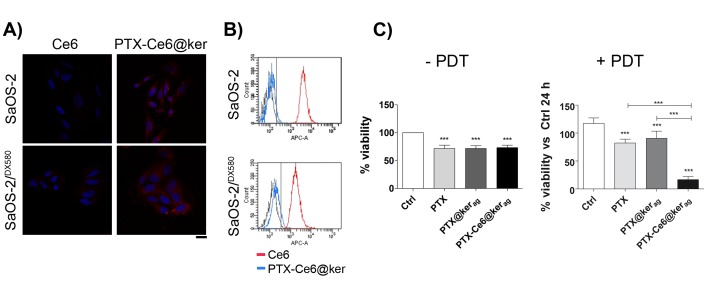
Impact of keratin nanoformulation on chemoresistant SaOS-2/^DX580^ cells. (**A**,**B**) SaOS-2 and SaOS-2^/DX580^ were treated for 24 h with Ce6 or PTX-Ce6@ker at a [Ce6] concentration of 3.35 µM. (**A**) Representative confocal microscopy images of cells treated with Ce6 or PTX-Ce6@ker_ag_. Scale bar: 25 µm. (**B**) the graphs show the Ce6 fluorescence after internalization of the photosensitizer by itself (blue line) or loaded into keratin nanoparticles (red line) quantified by flow cytometry analysis (Control, black line). (**C**) the graphs show the Alamar blue assay on SaoOS-2^/DX580^ after 24 h treatment with PTX, PTX@ker_ag_, or PTX-Ce6@ker_ag_ at an equivalent concentration of [PTX] of 13.4 µM (High) and 24 h after irradiation (+PDT). All data are normalized to untreated cells (Ctrl) and expressed as the mean ± SD (from at least two independent experiments performed in triplicate) and analyzed using a one-way ANOVA test, and Tukey’s multiple comparison test as a post-test. Results were considered to be statistically significant at *p* values < 0.05 (*** *p* values < 0.001).

Next, the efficacy of our keratin-based drug delivery system was tested on the SaOS-2^/DX580^ chemoresistant cell line. We first evaluated the advantages of using keratin for the delivery of Ce6, and then compared the results obtained on SaOS-2^/DX580^ with the parental cell line (SaOS-2). Fluorescent imaging (Figure 5A) shows that, in both cell lines, there is a low intracellular signal when free Ce6 (red signal) is administered, while the signal increases when Ce6 is vehiculated through keratin (PTX-Ce6@ker_ag_) at the same dosage as the free Ce6. These results were confirmed by flow-cytometry analyses (Figure 5B).

In order to evaluate the effect of PTX, alone or combined with keratin, and the potential additive effect of PDT and chemotherapy, SaOS-2/^DX580^ cells were treated with PTX, PTX@ker_ag_, and PTX-Ce6@ker_ag_, at high PTX concentration (Figure 5C −/+PDT). The same experiment was performed also at medium dosage (Figure S7). The viability was significantly affected when tumor cells were loaded with free PTX or PTX vehiculated by keratin, both in the presence or absence of Ce6. More importantly, OS cells loaded with the multi-modal nanoparticle formulation upon light irradiation (PTX-Ce6@ker_ag_ +PDT) showed a dramatic decrease in cell viability, demonstrating that, similarly to SaOS-2 cells, chemotherapy and photoactivation act in an additive manner in drug-resistant OS cells (Figure S6 +PDT). Interestingly, when SaOS-2^/DX580^ are treated with the medium concentration of PTX alone or combined with keratin nanoparticles, the nanoformulation improves the chemotherapeutic drug effect. However, at this dosage, the additive effect of chemo and photodynamic therapy upon PTX-Ce6@ker_ag_ +PDT is less prominent (Figure S7).

These results, together with the data reported in Figure 4 and Figure S6, show that the combination of chemotherapy and photoactivation (PTX-Ce6@ker_ag_ +PDT) result in massive cell death in all the cell lines tested, though most importantly on the chemoresistant-p-glycoprotein overexpressing SaOS-2 cells. This evidence is of particular relevance, since the overexpression of p-glycoprotein is one of the most common mechanisms of resistance against taxanes and one of the main causes of failure in second-line chemotherapy in relapsed OS patients.

### 2.6. In Vitro Toxicity of Keratin Nanoformulations in 3D System

**Figure 6:**
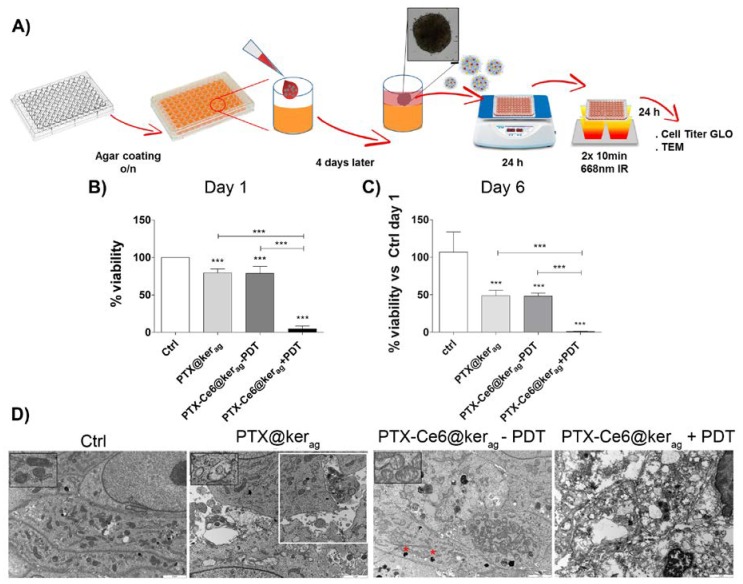
Impact of keratin nanoformulation on 3D OS tumor model. (**A**) Schematic representation of cell spheroid formation and treatment with PTX-Ce6@ker_ag_ at a [Ce6] concentration of 6.7 µM; (**B**, **C**) the graphs show the Cell Titer Glo assay performed 1 day (**B**) or 6 days (**C**) after keratin nanoparticle treatment and −/+ irradiation (−/+PDT). The results are normalized to Ctrl at day 1 (no treated cells) and expressed as mean ± SD (N = 6 technical replicates and *N* = 2 individual replicates experiments) using the one-way ANOVA test, and Tukey’s multiple comparison test as a post-test. Results were considered to be statistically significant at *p* values < 0.05. * *p*-values < 0.05, ** *p*-values < 0.01 and *** *p*-values < 0.001; (**D**) transmission electron microscopy of treated MG63 spheroids with PTX@ker_ag_ and PTX-Ce6@ker_ag_ one day after irradiation (+PDT). The black squares highlight the mitocondrial alterations after nanoparticle treatment, compared to Ctrl spheroids. The white square in PTX@ker_ag_ highlights the necrosis area, and the red star in PTX-Ce6@ker_ag_ -PDT highlights the vesicle containing semidigested keratin nanoparticles. Scale bar: 2 µm.

To confirm the results obtained in the 2D experiments, a three-dimensional (3D),. spheroid-based OS tumor model and a protocol for nanoparticles administration were developed (Figure 6A).

In the spheroids treated with a high dosage of PTX@ker, we observed a 20% cell viability reduction on day 1, which increases up to 50% after 6 days, thus confirming that the vehiculation of PTX through keratin was efficient at killing tumor cells in a three-dimensional environment as well (Figure 6B PTX@ker_ag,_). A similar result was obtained in the spheroids treated with multi-modal nanoparticles which were not irradiated (PTX-Ce6@ker_ag_-PDT, Figure 6B). The 50% drop can be ascribed to the prolonged effect of PTX on tumor spheroids, which results in the formation of necrotic area and the appearance of apoptotic nuclei, as revealed by the TEM images (Figure S8). Upon light photoactivation, the multi-modal nanoformulations induce more than a 95% decrease in cell viability. This percentage further drops after 6 days from light irradiation (PTX-Ce6@ker_ag_ +PDT, Figure 6C).

Ultrastructural analysis, performed at day 1 on spheroids treated with PTX@ker_ag_ (Figure 6D), displays an alteration of mitochondria morphology characterized by swelling, cristolysis, and rarefacted matrix (magnification), and an increase of necrotic area (white square) compared to untreated spheroids (Ctrl), where alterations which can be associated to PTX treatment. In PTX-Ce6@ker_ag_ with no light irradiation (−PDT), black vesicles/vacuoles immediately under the cell membrane are visible (red star), which is probably due to partially digested keratin nanoparticle aggregates. When irradiated (+PDT), massive necrosis can be observed inside the spheroids as a result of the activation of necrotic pathways, induced by ROS damage.

The 3D tumor model system also confirms the efficacy of our approach in a three-dimensional environment. Keratin nanoparticles are able to penetrate inside the cell mass, preserving the activity of both drugs. PTX alters the structure of mitochondria, causing a loss of matrix and cristolysis, as previously reported by others as a consequence due to the long exposure (24 h) to high PTX concentrations [47]. PTX acts on bcl-2 proteins activating caspases, and also alters the interaction between microtubules and the outer membrane of mitochondria. This leads to a dysfunction of PTP, and the consequent release of Ca^2+^ and cytochrome c into the cytoplasm [48,49]. A dysfunction of the mitochondria induces a significant reduction of ATP production, as also shown by the imaging and cell viability metabolic-based assays performed on the sixth day post-treatment.

## 3. Experimental Section

### 3.1. Materials

Dulbecco’s Modified Eagle’s Medium-high glucose (DMEM-HG, glucose 4500 mg/L), McCoy’s medium, WST-1 (Roche), Triton X-100, *N*-hydroxysuccinimide (NHS), 1-ethyl-3-(3-dimethyl-aminopropyl) carbodiimide (EDC), Phosphate Buffered Saline (PBS) were purchased by Sigma Aldrich (Saint Luis, MI, USA). Alamar Blue, WST-1, CellTiter-GLO^®^ were purchased from Promega (Milano, Italy).

Iscove’s modified Dulbecco’s Medium (IMDM), Fetal Bovine Serum (FBS), Fetal Calf serum (FCS), GlutaMAX, penicillin/streptomycin. Dulbecco’s Phosphate buffered saline (D-PBS)-, Alamar Blue, were purchased from Thermo Fisher Scientific (Waltham, Massachusetts, USA).

MG63 (CRL-1427), U-2 OS (HTB-96), and SaOS-2 (HTB-85) cell lines were purchased from ATCC (Manassas, Virginia, USA). Cells were cultured respectively in DMEM-HG (MG63) or McCoy’s medium (U-2 OS and SaOS-2) containing 10% of FBS, 1% GlutaMAX, and 50 U/mL penicillin/streptomycin, at 37 °C in a humidified atmosphere of 5% CO_2_ air.

SaOS-2/^DX580^ [50] were cultured in IMDM supplemented with 10% of inactivated FCS 50 U/ml penicillin/streptomycin, at 37 °C in a humidified atmosphere of 5% CO^2^ air.

Primary antibodies used were: mouse β-tubulin (1:50) (Sigma Aldrich), mouse anti-EEA1 (1:100) (Thermo Fisher); anti-Lamp1/CD107a (1:1000) (Thermo Fisher).

The secondary antibody used was: anti-mouse IgG-Alexa 555 (1:200) (ThermoFisher). Actin staining: Phalloidin-FITC (1:100) (Sigma Aldrich). Lysosomes staining: Lysotracker DND26 (50 nM) (Thermo Fisher).

Hydrosoluble keratin was kindly provided by Kerline Srl (Bologna, Italy). PTX was purchased by TCI-Europe (Zwijndrecht, Belgium). Chlorin-e6 was purchased from Livchem Logistics GmbH (Frankfurt am Main, Germany).

### 3.2. Keratin Nanoformulations Synthesis

PTX-Ce6@ker synthesis by the drug-induced aggregation method (PTX-Ce6@ker_ag_) was prepared as follows: a PBS solution of Ker-Ce6 and pristine keratin was prepared with a final Ce6 concentration of 40 µg/mg_ker_ and a final keratin concentration of 5 mg/mL_PBS_. The solution was then sonicated for 30 min in a refrigerated bath (20 °C), and a solution of PTX (10% w_PTX_/w_ker_) in ethanol (10 mg/mL) was slowly added (0.3 mL/min) via syringe pump and under vigorous stirring (600 rpm). The obtained solution was then stirred for an additional hour, checked by dynamic light scattering, and lyophilized to afford a powder of PTX-Ce6@ker_ag_.

PTX-Ce6@ker synthesis by the desolvation method (PTX-Ce6@ker_ds_) was prepared as follows: Ker-Ce6 (7.31 mg) was dissolved in NaHCO_3_ buffer (500 µL) at room temperature in the dark for 1 h. 91.5 µL of a PTX solution in ethanol (10 mg/mL) was diluted with ethanol (4 times buffer volume) and slowly added to the mixture. After protein desolvation, 2.92 µL of glutaraldehyde, 8% (0.4 µL/mg keratin) was added for particle stabilization by cross-linking. The suspension of keratin nanoparticles was purified by 4 cycles of centrifugation (12,000 rpm) for 15 min with filter devices (MWCO: 100 kDa) to remove ethanol, unreacted glutaraldehyde, and free keratin. For the first cycle, particles were re-suspended with 500 µL of water/ethanol mixed solution (80/20), while for the following cycles, only water was used (500 µL).

PTX@ker was prepared by both the desolvation (PTX@ker_ds_) and aggregation method (PTX@ker_ag_), while Ce6@ker was prepared exclusively by using the desolvation method [39].

### 3.3. Keratin Nanoformulations Characterization

Loading of the Ce6 onto keratin was evaluated by recording the absorption spectra of the Ker-Ce6 suspension with a UV-Vis spectrophotometer Cary 100 (Agilent Technologies). For Ce6 quantification, a calibration curve of Ce6 dissolved in NaHCO_3_ buffer (pH = 9.2) in the 0–5 mg/mL concentration range was determined (Figures S1 and S2). PTX-loading onto nanoparticles was evaluated by UV-vis measurements through a calibration curve obtained on the PTX absorbance peak at 230 nm (Figure S3). Nanoparticle morphology was analyzed by Transmission Electron Microscopy (TEM) (for further details see paragraph below).

The hydrodynamic diameter in aqueous solutions (0.5 mg/mL) was determined by photon correlation spectroscopy (PCS) at 25 °C, using a NanoBrook Omni Particle Size Analyzer (Brookhaven Instruments Corporation, New York, NY, USA) equipped with a 35 mW red diode laser (nominal 640 nm wavelength). As far as the electrophoretic mobility is concerned, zeta-potential was measured at 25 °C by means of the same system.

PTX-Ce6@ker stability in physiological conditions was determined by dissolving 500 µg of nanoparticles at a medium concentration of both drugs—for example, [Ce6] = 3.35 µM and [PTX] = 6.52 µM, in 2 mL of PBS and maintaining them at 37 °C. The size of the keratin nanoparticles and polydispersity over time was checked by dynamic light-scattering analysis at pre-determined time intervals.

### 3.4. PTX Release

The evaluation of PTX release from PTX-Ce6@ker_ds_ was performed as follows: 6.3 mg of PTX-Ce6@ker_ds_ (lyophilized powder) containing 411 µg of PTX was diluted with 2 mL of PBS, inserted in a dialysis bag (cut-off 12 kDa) and dialyzed against a solution of PBS/EtOH (10 mL; 10% of EtOH). The system was heated at 37 °C while being stirred for 1 h (×3) and 2 h (×2) until reaching 24 h. Periodically, the outer solution was withdrawn, while fresh buffer solution was added back to the incubation media. At each time-point, the PBS/EtOH solution was extracted with CH_2_Cl_2_ (×3), and the collected organic phases were dried over Na_2_SO_4_ and concentrated under vacuum. The residue was dissolved in absolute EtOH (1 mL) and analyzed by UV-Vis spectroscopy (230 nm) and compared with a PTX calibration curve which was previously recorded (Figure S4). The same procedure was applied for determining PTX release from PTX-Ce6@ker_ag_, starting from 5.6 mg of nanoparticle powder, loaded with 505 µg of PTX.

### 3.5. In Vitro Toxicity in 2D

For IC_50_, OS cells were seeded 5 × 10^3^ onto a 96-well plate and treated for 24 h with the nanoformulations. According to the OS cells’ kinetics of growth [51], WST-1 was performed according to the manufacturer’s protocol after 48 h (MG63 and U2-OS) and 5 days (SaOS-2) from loading. The IC_50_ value was calculated by applying a sigmoidal dose response function on the results of three independent experiments in GraphPad PRISM software (vs 6, GraphPad Software, La Jolla, CA, USA).

For in vitro toxicity, OS cell lines were seeded 10^4^ cells/cm^2^ onto 24-well plates and treated for 24 h with PTX-Ce6@ker_ag_ (Table 1). Cells were then washed twice with D-PBS, and a new complete medium was added to each well. Plates were then exposed to LED light at 668 nm ± 3 nm for 5 min (fluence 163 J/cm^2^). An alamar blue assay was performed at the end of the treatment and 24 h after irradiation, according to the manufacturer’s protocol.

### 3.6. Cellular localization analyses

The cellular uptake of Ce6 or Ce6@ker was evaluated by a flow cytometer, where we used the FACScanto II cytometer (Becton-Dickinson, Franklin Lakes, New Jersey, USA) and Nikon TiE microscope equipped with a fully automated A1 confocal laser which incorporates the resonant scanner with a resonance frequency of 7.8 kHz which allows high-speed imaging (A1R, Nikon, Amsterdam, Netherlands), and equipped with DS-QiMc-U2 12 bit camera. using 60× oil plan Fluo objection NA: 1.4 (Nikon, Amsterdam, the Netherlands). Immunofluorescence was performed as previously described [38]. The colocalization of the fluorochromes was evaluated using Pearson’s colocalization coefficient [52]. To quantify the multi-lobated nuclei, ten different images for each condition (untreated cell, Ce6@ker- or PTX-Ce6@kerag-treated) were acquired using confocal microscopy. The number of total and multi-lobated nuclei were counted using NIS element software (Nikon Instruments Europe B.V., Nikon, Amsterdam, The Netherlands).

### 3.7. In Vitro Toxicity in 3D

To induce spheroid formation, MG63 suspension at a concentration of 2 × 10^4^ cells/100µL was dispensed onto a 96-well plate previously coated with 3% agar in PBS [53] and left to aggregate for three days. Spheroids were treated with a high concentration (see Table 1) of Ce6@ker, PTX@ker_ag_, or PTX-Ce6@ker_ag_ for 24 h under continuous agitation. At the end of the treatment, the media containing the nanoparticles was removed, spheroids were washed twice with PBS, and half of them were subsequently irradiated for 10 min using 668 nm LED light (fluence 336 J/cm^2^). CellTiter-GLO^®^ was performed one day and six days later, according to the manufacturer’s protocol.

### 3.8. Transmission Electron Microscopy (TEM)

Keratin nanoparticles at a concentration of 1.06 µg/µL were dispensed by droplets onto a formvar grid, and allowed to dry for 1 h. Keratin nanoparticles were fixed in 2.5% glutaraldehyde in cacodylate buffer 0.1 M, and washed three times in water.

MG63 OS tumor spheroids were fixed and processed as previously described [54], and subsequently observed with a Jeol Jem-1011 transmission electron microscope (Jeol Peabody; Peabody, MA, USA).

### 3.9. Statistical Analysis

All results are expressed as the mean ± SD (from at least two independent experiments performed in triplicate) and analyzed using the one-way ANOVA test, and Tukey’s multiple comparison test as a post-test. Results were considered to be statistically significant at *p* values < 0.05. The data were processed with GraphPad Prism 5 software (GraphPad; San Diego, CA, USA).

## 4. Conclusions

Considering the vast heterogeneity of OS’ molecular profiles and the standard chemotherapeutic treatment limits, in our study we propose a treatment that moves away from targeted anti-oncogenic paradigms toward less toxic drug formulation, and more generalized oxidative-damage approaches. In this paper, we demonstrated, for the first time, the efficacy of a novel, bimodal, keratin-based nanoformulation that combined chemotherapy and photoactivation (PTX-Ce6@ker_ag_ +PDT) both in 2D and 3D osteosarcoma models. This study demonstrated that the combination of PTX and PDT was also highly effective for drug-resistant SaOS-2/^DX580^ cells. It is worthwhile noting that these cells are prone to overexpressing p-glycoprotein, which is the clinically most relevant mechanism of resistance described in high-grade OS [55], and is also responsible for PTX unresponsiveness. Therefore, this multi-modal combined approach appears to be a new, promising therapeutic strategy that may improve treatment efficacy and prognosis, first of all in relapsed OS patients.

Overall, our results serve as important preliminary data that will allow us to test their efficacy in vivo, which is a necessary step toward clinical translation. If the nanoformulation proves to be effective in relevant preclinical and clinical trials, we will contribute to providing more efficient pharmacological treatment, and increase the current overall survival rate of OS-affected patients.

## Figures and Tables

**Table 1:** Values of the three different concentrations of PTX-Ce6@ker_ag_ nanoparticles, defined as PTX-Ce6@ker_ag_ (*low*), PTX-Ce6@ker_ag_ (*medium*), and PTX-Ce6@ker_ag_ (*high*).

Entry	Concentration/Nanoparticles	Low	Medium	High
1	[Ce6] in PTX-Ce6@ker_ag_	0.84 µM	3.35 µM	6.7 µM
2	[PTX] in PTX-Ce6@ker_ag_	1.63 µM	6.52 µM	13.0 µM

## Supplementary Materials

Supplementary materials can be found at http://www.mdpi.com/1422-0067/19/11/3670/s1.
