# Supplementary material for: Functionalized Keratin as Nanotechnology-Based Drug Delivery System for the Pharmacological Treatment of Osteosarcoma
Source: Int J Mol Sci. 2018 Nov 20;19(11):3670. doi: 10.3390/ijms19113670 (PMC6274803; doi:10.3390/ijms19113670)
Supplement: Supplementary file 1 [file ijms-19-03670-s001.pdf]

Copyright WILEY-VCH Verlag GmbH & Co. KGaA, 69469 Weinheim, Germany, 2016.

Supporting Information

Functionalized Keratin as Nanotechnology-Based Drug Delivery System for the Pharmacological Treatment of Osteosarcoma

Elisa Martella, Claudia Ferroni, Andrea Guerrini, Marco Ballestri, Marta Columbaro, Spartaco Santi, Giovanna Sotgiu, Massimo Serra, Davide Maria Donati, Enrico Lucarelli, Greta Varchi and, Serena Duchi*

Tables.

Entry	Effective Diam. (nm)	Polydispersity	Baseline Index	Zeta-potential (mV)	Mobility (μ/s)/(V/cm)
1	157,79	0.199	7,8	-46,77	-3,8
2	154,36	0,213	7,8	-45,32	-3,72
3	152,85	0,227	9,3	-47,01	-3,57
4	151,54	0,225	7,9	-44,25	-3,79
5	151,87	0,205	9,4	-44,91	-3,48
Mean	153,68	0,214	8,5	-45,65	-3,67
Std Dev	2,54	0,012	0,8	1,20	0,14

Table S1. **PTX-Ce6@ker$_{ag}$** hydrodynamic diameters.

Entry	Effective Diam. (nm)	Polydispersity	Baseline Index	Zeta-potential (mV)	Mobility (μ/s)/(V/cm)
1	123,84	0,073	8,6	-49,92	-3,90
2	122,40	0,088	9,7	-46,80	-3,66
3	121,10	0,067	8,5	-47,59	-3,73
4	121,06	0,059	9,8	-45,93	-3,59
5	121,67	0,047	9,2	-46,45	-3,63
Mean	122,01	0,067	9,2	-47,34	-3,70
Std Dev	1,16	0,015	0,6	1,56	0,12

Table S2. **PTX-Ce6@ker$_{ds}$** hydrodynamic diameters.

Cell Line	PTX	PTX@ker$_{ag}$	PTX-Ce6@ker$_{ag}$	PTX@ker$_{ds}$	PTX-Ce6@ker$_{ds}$
MG63	1.06x10^{-10} M	2,79x10^{-10} M	1.26x10^{-10} M	2,61x10^{-8} M	6,16x10^{-8} M
SaOS-2	7,95 x10^{-10}M	1,08 x 10^{-9} M	1,453 x 10^{-9} M	9,6 x 10^{-8} M	1 x 10^{-7} M
U-2 OS	5,672x10^{-10} M	1,59x10^{-9} M	5,716x10^{-10} M	0,372x10^{-6} M	0,297 x10^{-6} M

Table S3. IC50 values of PTX, PTX@ker$_{ag/ds}$ and PTX-Ce6@ker$_{ag/ds}$ on MG63, SaOS-2 and U-2 OS cells (N= 3 technical replicates and N=3 individual replicates experiments).

Figures.

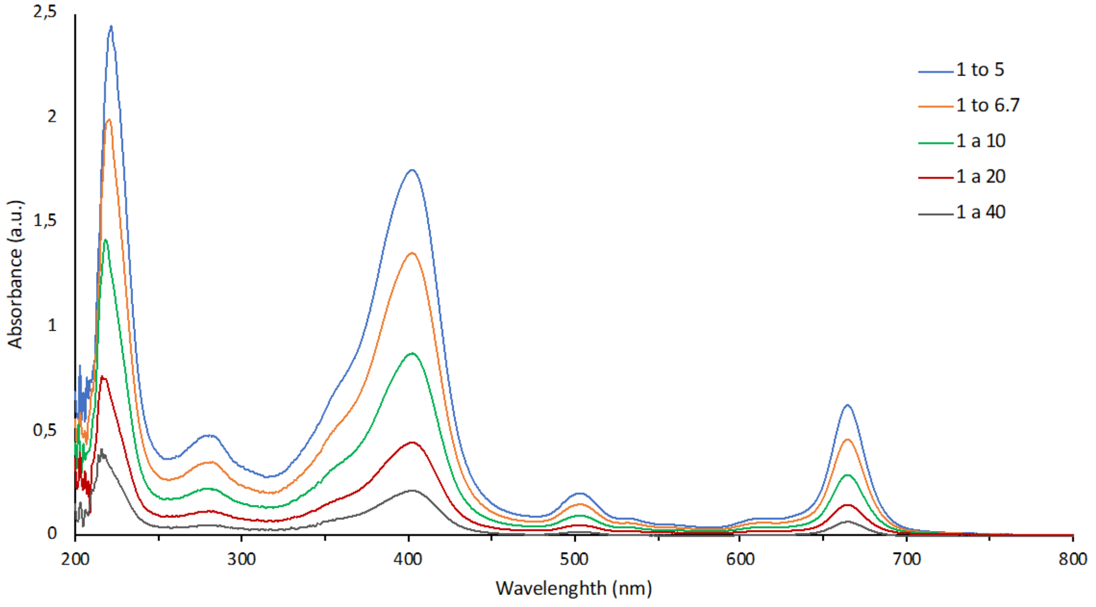

Figure S1. Absorbance spectra of chlorin-e6 solution at different Ce6 concentrations.

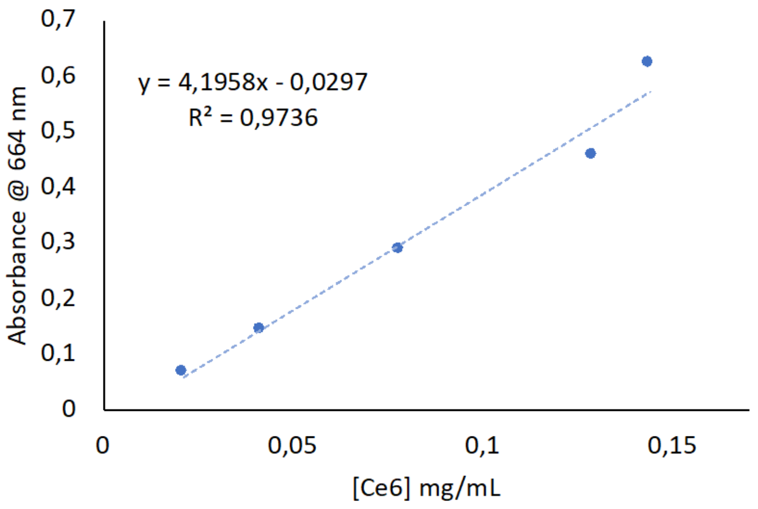

Figure S2. Chlorin-e6 calibration curve.

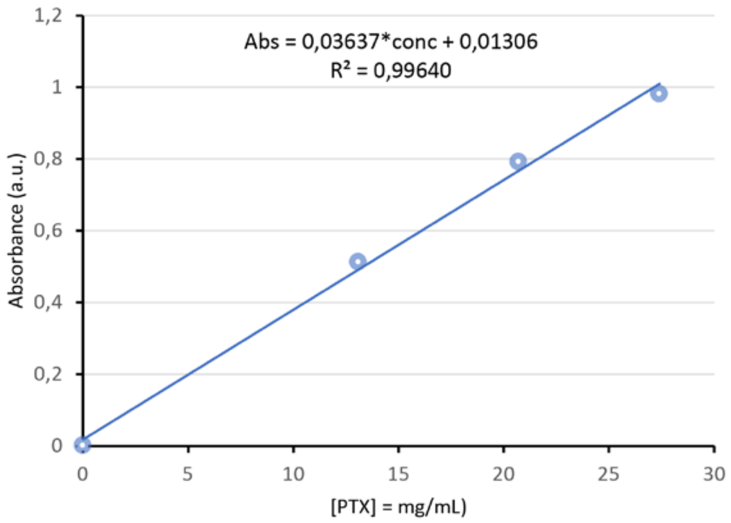

Figure S3. PTX calibration curve.

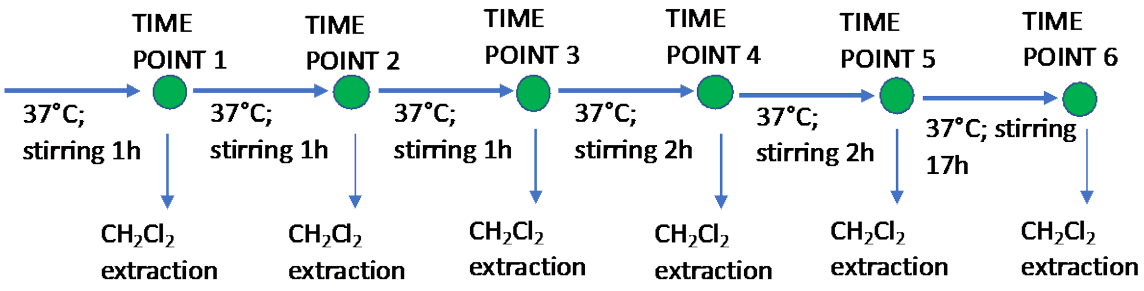

Figure S4. Graphical flow chart of the protocol used to evaluate PTX release over time.

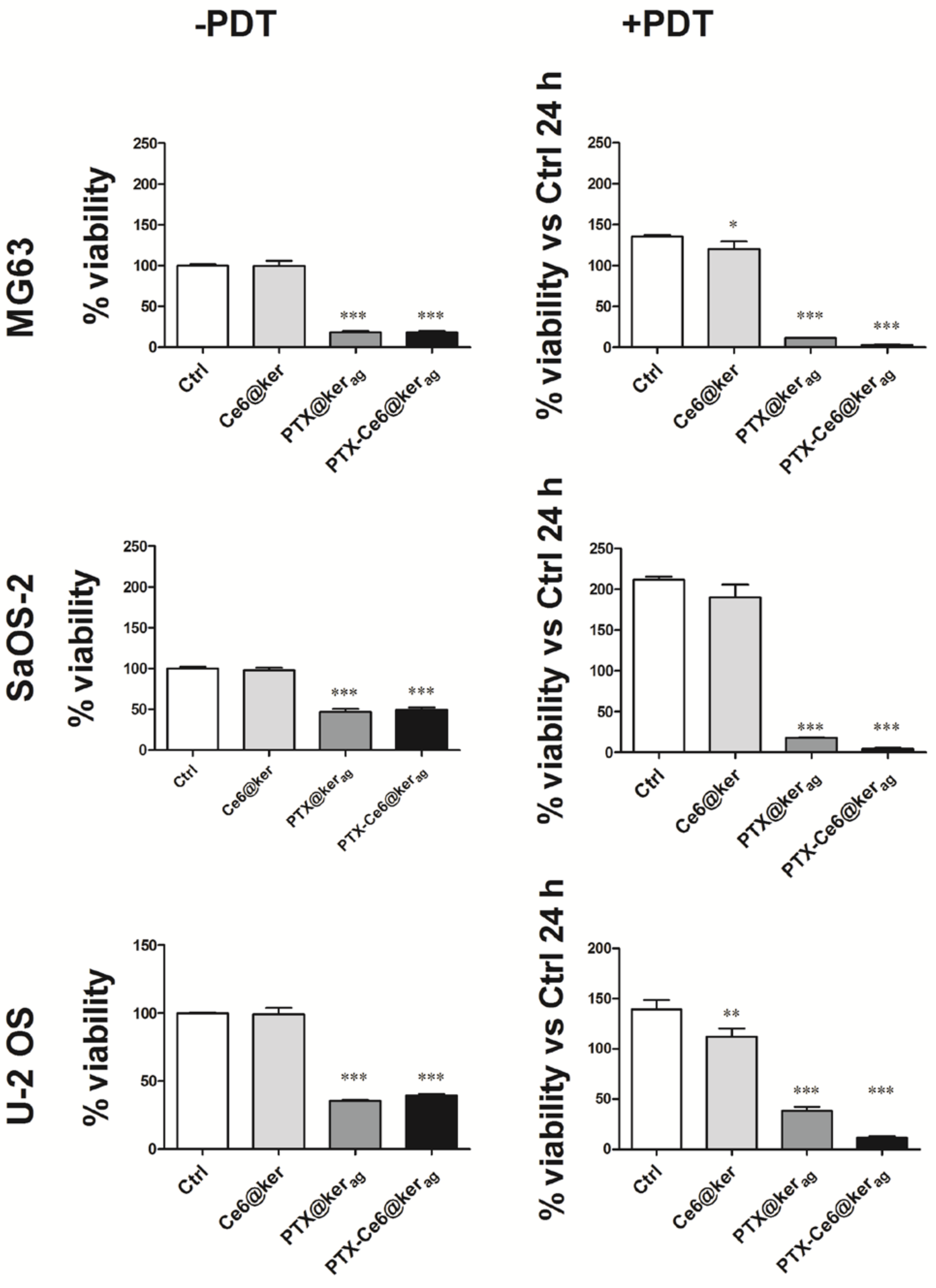

Figure S5. OS cell lines were treated for 24 h with Ce6@ker, PTX@ker_{ag} or PTX-Ce6@ker_{ag} at low dosages (Ce6 0,84 µM and PTX 1,63µM). Alamar blue was performed

immediately after Keratin nanoparticles treatments (- PDT), and 24 h after irradiation of the same samples (+ PDT). Data, normalized to not treated cells (Ctrl) at first time point, are expressed as the mean±SD (N=2 biological replicates; N= 3 technical replicates) and analyzed using 1-way ANOVA test and Tukey's multiple comparison test as a post test. Results were considered to be statistically significant at P-values < 0.05. *P-values < 0.05, **P-values < 0.01 and ***P-values < 0.001

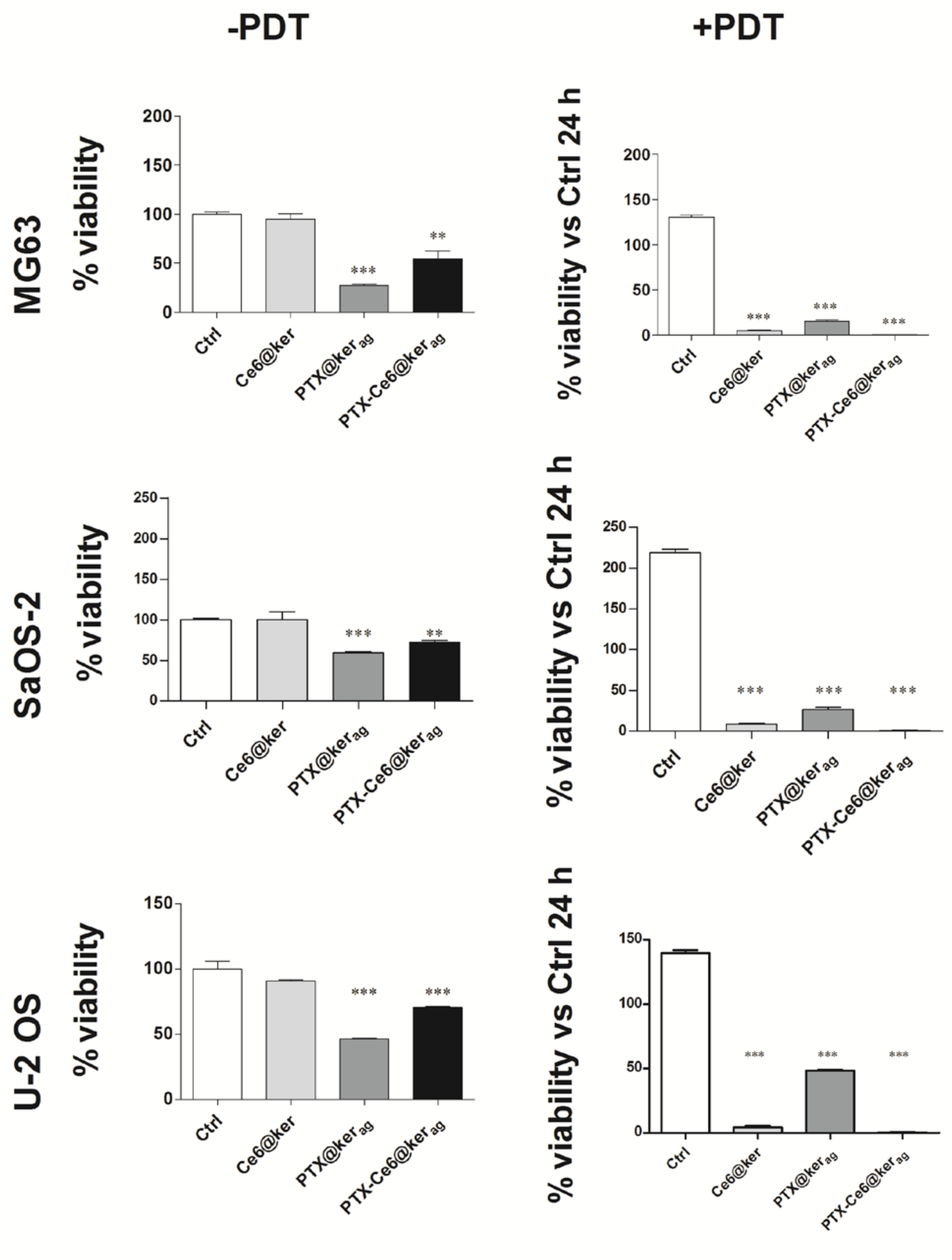

Figure S6. OS cell lines were treated for 24 h with Ce6@ker, PTX@ker$_{ag}$ or PTX-Ce6@ker$_{ag}$ at High dosages (Ce6 6,7 µM and PTX 13.4µM). Alamar blue was performed immediately after Keratin nanoparticles treatments (- PDT), and 24 h after irradiation of the same samples (+ PDT). Data, normalized to not treated cells (Ctrl) at first time point, are expressed as the mean±SD (N=2 biological replicates; N= 3 technical replicates) and

analyzed using 1-way ANOVA test and Tukey's multiple comparison test as a post test. Results were considered to be statistically significant at P-values < 0.05. **P-values < 0.01 and ***P-values < 0.001.

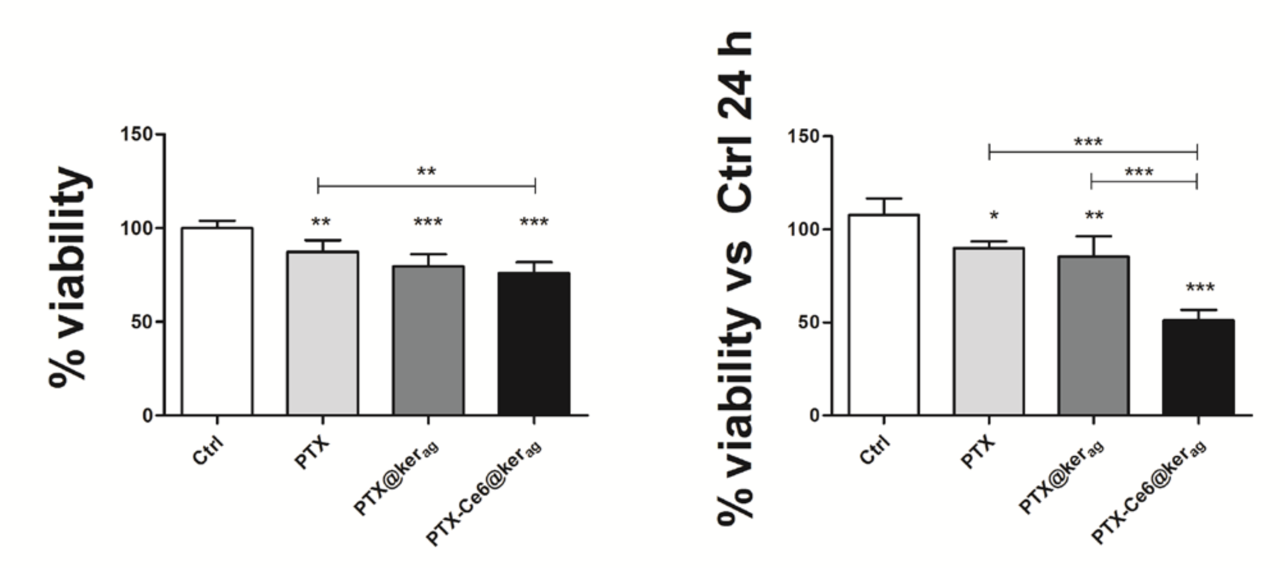

Figure S7. Alamar blue assay on SaoOS-2/^{DX580} after 24 h treatment with PTX, PTX@ker$_{ag}$ or PTX-Ce6@ker$_{ag}$ at an equivalent concentration of [PTX] of 6.7µM (Medium) and 24 h after irradiation (+PDT). All Data are normalized to not treated cells (Ctrl) and expressed as the mean ±SD (from at least two independent experiments performed in triplicate) and analyzed using 1-way ANOVA test and Tukey's multiple comparison test as a post test. Results were considered to be statistically significant at P-values < 0.05. **P-values < 0.01 and ***P-values < 0.001.

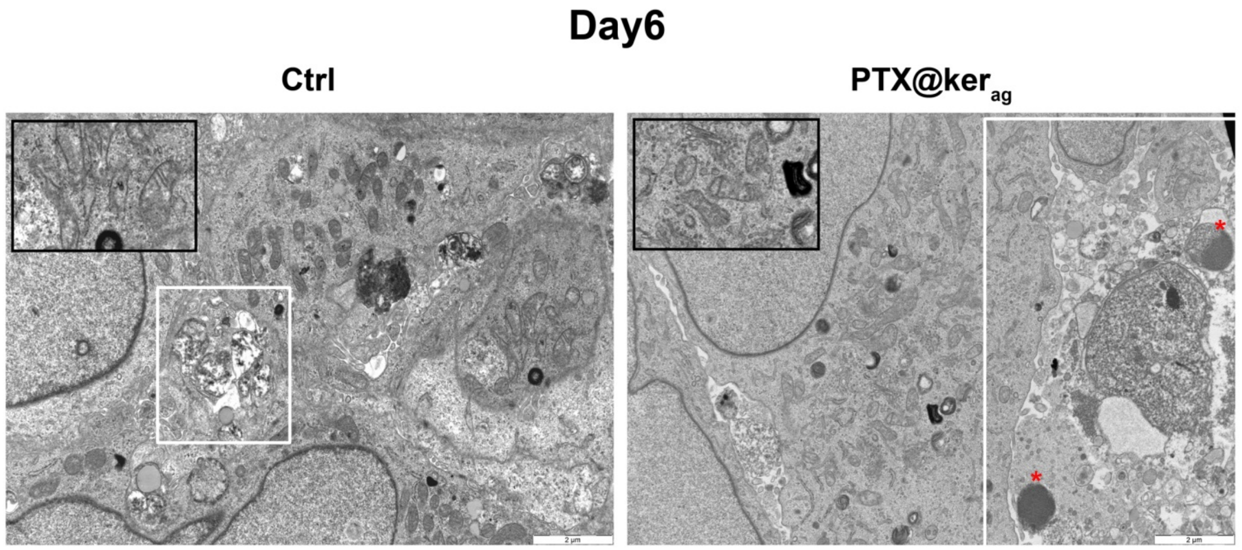

Figure S8. Transmission electron microscopy was performed on MG63 spheroids six days after PTX@ker$_{ag}$ treatment with no light activation. White squares indicate necrotic and autophagic area and red stares highlight apoptotic nuclei.
